# Modeling the effect of age on quantiles of the incubation period distribution of COVID-19

**DOI:** 10.1186/s12889-021-11761-1

**Published:** 2021-09-27

**Authors:** Xiaohui Liu, Lei Wang, Xiansi Ma, Jiewen Wang, Liwen Wu

**Affiliations:** 1 School of Statistics, Jiangxi University of Finance and Economics, No.169, East Shuanggang Road, Nanchang, Jiangxi Province, 330013 China; 2 Research Center of Applied Statistics, Jiangxi University of Finance and Economics, No.169, East Shuanggang Road, Nanchang, Jiangxi Province, 330013 China; 3 National Engineering Laboratory for Educational Big Data, Central China Normal University, NO.152 Luoyu Road, Wuhan, Hubei Province, 430079 China; 4 Department of Neurology, Hunan children’s Hospital, No.86 Ziyuan Road, Changsha, Hunan Province, 410000 China

**Keywords:** COVID-19, Incubation period, Conditional quantiles, Biased sampling

## Abstract

**Background:**

The novel coronavirus SARS-CoV-2 (coronavirus disease 2019, COVID-19) has caused serious consequences on many aspects of social life throughout the world since the first case of pneumonia with unknown etiology was identified in Wuhan, Hubei province in China in December 2019. Note that the incubation period distribution is key to the prevention and control efforts of COVID-19. This study aimed to investigate the conditional distribution of the incubation period of COVID-19 given the age of infected cases and estimate its corresponding quantiles from the information of 2172 confirmed cases from 29 provinces outside Hubei in China.

**Methods:**

We collected data on the infection dates, onset dates, and ages of the confirmed cases through February 16th, 2020. All the data were downloaded from the official websites of the health commission. As the epidemic was still ongoing at the time we collected data, the observations subject to biased sampling. To address this issue, we developed a new maximum likelihood method, which enables us to comprehensively study the effect of age on the incubation period.

**Results:**

Based on the collected data, we found that the conditional quantiles of the incubation period distribution of COVID-19 vary by age. In detail, the high conditional quantiles of people in the middle age group are shorter than those of others while the low quantiles did not show the same differences. We estimated that the 0.95-th quantile related to people in the age group 23 ∼55 is less than 15 days.

**Conclusions:**

Observing that the conditional quantiles vary across age, we may take more precise measures for people of different ages. For example, we may consider carrying out an age-dependent quarantine duration in practice, rather than a uniform 14-days quarantine period. Remarkably, we may need to extend the current quarantine duration for people aged 0 ∼22 and over 55 because the related 0.95-th quantiles are much greater than 14 days.

**Supplementary Information:**

The online version contains supplementary material available at (10.1186/s12889-021-11761-1).

## Background

In December 2019, some cases of pneumonia with unknown etiology were identified in Wuhan, Hubei province in China. After being investigated by the National Coronavirus Research Group, this pneumonia was identified as caused by a new coronavirus (2019-nCoV). The World Health Organization (WHO) has named this disease COVID-19, standing for “2019 coronavirus disease” [1].

It turns out that the novel coronavirus, like SARS-COV, is the seventh member of the Nidovirales family of coronaviruses [2], but COVID-19 has a shorter serial interval than that of SARS [3] and higher transmissibility than MERS in the Middle East countries [4]. It is highly infectious [5] and even contagious during the incubation period [6]. It can cause severe symptoms or even death [7]. The novel coronavirus not only threatens cities in China [8], but also seems to have exploded worldwide. Hence, it is important to take necessary measures to prevent and control it as quickly as possible.

In prevention and control efforts, it is well known that the incubation period distribution plays an important role. Knowledge of this distribution can help mathematically model the size of the epidemic [8], predict the time at which the disease will outbreak, and determine the efficacy of the medical intervention [9], etc. The pioneering work on deriving the incubation period distribution was conducted by Philip Sartwell in 1950 [10]. After that, the lognormal distribution was widely used to model the incubation period distribution for infectious diseases. Many authors studied the incubation period distributions of various other diseases. Some other distributions, e.g., Gamma distribution and Weibull distribution, were also suggested to fit the observed incubation periods; see for example [9, 11–13].

In the literature, Li et al. [14] first studied the incubation period distribution of COVID-19 based on the early 10 observations in Hubei province in China. Relying on their estimation, Li et al. [14] suggested a 14-day medical observation period or quarantine for exposed persons. Guan et al. [15] reported the median incubation period, i.e., 3.0 days (range 0 to 24.0 days), of 1099 patients from 552 hospitals in 31 provinces/provincial municipalities through January 29th, 2020. Recently, Backer et al. [16] updated this distribution based on the reported travel histories and symptom onset dates of 88 travelers from Wuhan with confirmed 2019-nCoV infection in the early outbreak phase. Backer et al. [16] estimated that the 97.5 percentile of the incubation period distribution of COVID-19 is 11.1 days. Linton et al. [17] further considered the biased sampling issue and obtained that the estimated 95%-th quantile is greater than 14 days.

However, no existing literature above investigates the distributed characteristic of the incubation period of COVID-19 over people of different ages. Based on 2172 confirmed cases collected outside Hubei provinces in China, a simple ANOVA indicates that the age of confirmed cases has a significant effect on the incubation period of COVID-19 [18]. This motivates us to estimate the conditional incubation period distribution on ages. Note that the collected data subject to biased sampling because COVID-19 is still ongoing throughout February 16th, 2020 in China. The current study differs itself from [19–21], and [22], which investigated the relationship between the age and the incubation period of AIDS, but did not touch the biased sampling issue.

In this study, we developed the conditional quantiles model of the incubation period of COVID-19 on the age of infected cases and provided the estimating method in detail. The main results were calculated based on the collected data, and the conclusion was presented accordingly.

## Methods

In this section, we provide a summary on the collected data, and introduce the estimating method according to the major characters of the collected data.

**Fig. 1:**
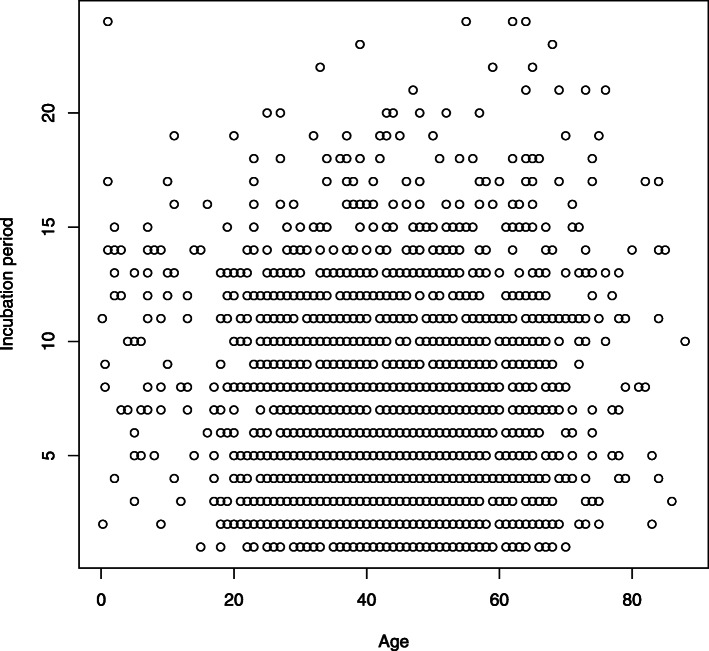
Scatter plot of the incubation period of COVID-19 versus the ages of confirmed cases. Note that many data points are overlapped

The data set is taken from the websites of the health commission, or the daily public reports on COVID-19 in 29 provinces outside Hubei province through February 16th, 2020. It consists of 2172 confirmed cases, including four indexes, i.e., gender, age, onset time, and infection time. The incubation period value here is calculated by using the formula “*Incubation Period* =*Onset date* −*Infection date* + 1”. Note that the default count unit is supposed to be ‘day’ throughout this paper. Among these 2172 cases, there are more cases from Zhejiang, Henan and Anhui than from the other provinces because of the large population of confirmed cases in these provinces. An additional file shows the details in the data (see Additional file 1). Figure 1 reports the scatter plot of the incubation period of COVID-19 v.s. the age of confirmed cases.

We conduct a preliminary one-way ANOVA study on the incubation period of COVID-19 over four age groups, i.e., 0 ∼17, 18 ∼40, 41 ∼65, and over 65, and find that the age of confirmed cases has a significant effect on the incubation period. Hence, we further investigate the incubation period distribution of COVID-19 conditional on age as follows.

Note that the Weibull distribution fits well the data set, and the mean incubation period varies over people of different ages. We propose to model the relationship between the true incubation period, say *T*, and the age, i.e., *X*, through the conditional distribution *G*(*t*|*λ*(*X*),*η*) on *X*. The related density function is specified as follows.

$$\begin{array}{@{}rcl@{}} g(t|\lambda(X),\eta)=\frac{\eta}{\lambda(X)}\left(\frac{t}{\lambda(X)}\right)^{\eta-1} \\ \exp\left(-\left(\frac{t}{\lambda(X)}\right)^{\eta}\right)I(t\ge 0), \end{array} $$

where *I*(·) denotes the indicator function, and *η*>0 and *λ*(*X*):=*λ*_3_(*X*)=***X***^⊤^***β***>0, where ***X***=(1,*X*,*X*^2^,*X*^3^)^⊤^, ***β***=(*β*_0_,*β*_1_,*β*_2_,*β*_3_)^⊤^.

The reasons for using this kind of conditional distribution form are as follows: (i) The conditional mean of *T* takes the form *E*(*T*|*X*)=*λ*(*X*)*Γ*(1+1/*η*), which implies that the age *X* has an obvious effect on *E*(*T*|*X*) through *λ*(*X*); (ii) *λ*_3_(*x*) is flexible enough to characterize the trend of the change of *E*(*T*|*X*) over *X*. Note that *λ*_3_(*x*) includes *β*_0_, *β*_0_+*β*_1_*x*, and *β*_0_+*β*_1_*x*+*β*_2_*x*^2^ as special cases. Here *Γ*(·) denotes the gamma function.

**Fig. 2:**
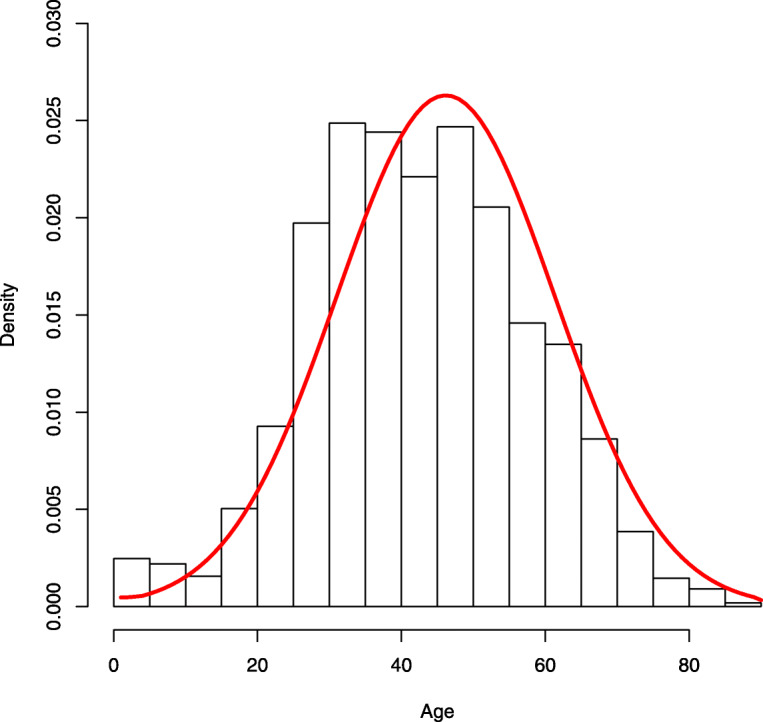
Histogram of the ages of confirmed cases. The line stands for the fitted density function of normal distribution

Furthermore, information from the empirical result shown in Fig. 2 indicates that one may model the distribution of the age *X* by normal distribution. Write its density as *ϕ*(*x*;*μ*,*σ*^2^). Then, a natural idea is through maximizing the likelihood function

1 $$\begin{array}{@{}rcl@{}} L_{T}(\boldsymbol{\beta}, \eta, \mu, \sigma^{2}) = \prod_{j=1}^{m} \left\{g(T_{j}|\lambda(X_{j}),\eta)\phi\left(X_{j}; \mu, \sigma^{2}\right)\right\}^{1/m} \end{array} $$

to estimate the conditional distribution based on $\{T_{j}, X_{j}\}_{j=1}^{m}$.

Unfortunately, the incubation period of some infected cases cannot be fully observed when the COVID-19 is still ongoing. The observed incubation period of COVID-19, say *Y*, subjects to biased sampling. That is, *Y* observed at some fixed time *t*_∗_ is not the same as the true incubation period *T*. This is because, for some case infected at time *t*_*S*_, we only can observe such incubation period *Y* with *Y*=*T* at time *t*_∗_ if *T*∈(0,*Δ*], where *Δ*=*t*_∗_−*t*_*S*_. This implies that the distribution of *Y* is in fact the conditional distribution depending on the random event {*T*∈(0,*Δ*]}. That is, we have

2 $$\begin{array}{@{}rcl@{}} F(y|X) &=& P(Y\le y|X)\\ &=& P(Y \le y| T \in (0, \Delta], X)\\ &=& \frac{P(T\le y, 0 < T\le \Delta|X)}{P(T \in (0, \Delta]|X)}\\ &=& \left\{ \begin{array}{cll} 0,& & \quad\text{if}\ y \le 0\\[0.8ex] \frac{G(y|\lambda(X), \eta)}{G(\Delta|\lambda(X), \eta)},&& \quad\text{if}\ 0 < y < \Delta\\[0.8ex] 1,& & \quad\text{if}\ y \ge \Delta. \end{array} \right. \end{array} $$

Denote the collected samples as $\{Y_{i}, \Delta _{i}, X_{i}\}_{i=1}^{n}$, where *Y*_*i*_’s denote the observed incubation periods, *X*_*i*_’s the ages, and *Δ*_*i*_=*t*_∗_−*t*_*S*,*i*_ the difference between the infected time of the *i*-case and the observing time *t*_∗_, i.e. February 16th, 2020.

**Fig. 3:**
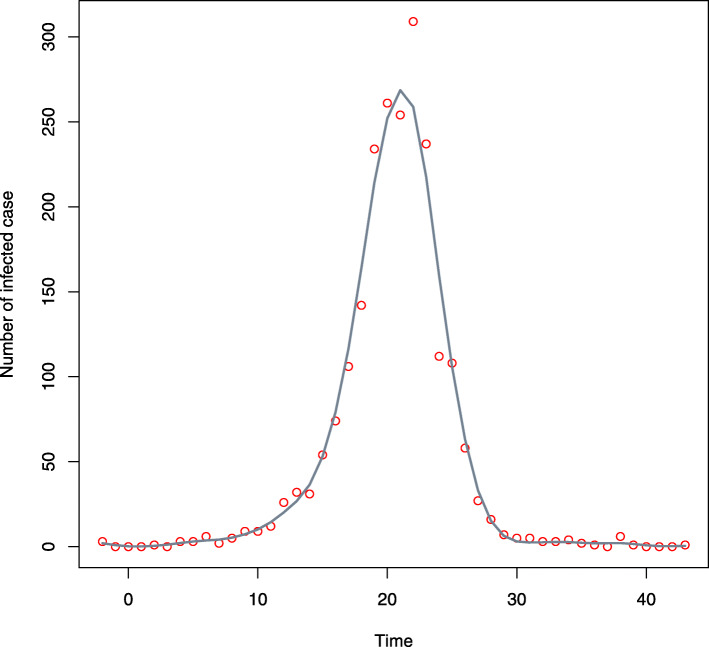
Scatter plot of the number of infected cases versus the infected time. The line stands for the fitted line through the least squares method

Since the number of infected cases does not grow exponentially throughout February 16th, 2020 (see Fig. 3), it is unreasonable to use the likelihood function developed in [17] again in this paper. Fortunately, note that there are cases infected almost every day throughout the data collecting time. Hence, it is reasonable to assume that *Δ*_*i*_’s are non-random. Furthermore, note that *Y*_*i*_,*X*_*i*_ are independent of the number of infected cases in each day *t*_*S*,*i*_. Then, after obtaining *F*(*y*|*X*) in (2), we propose to use the following likelihood function:

3 $$\begin{array}{@{}rcl@{}} &&\ell_{Y}(\boldsymbol{\beta}, \eta, \mu, \sigma^{2}) \\ &= &\prod_{i=1}^{n} \Bigg\{\left[g(Y_{i}| \lambda(X_{i}), \eta)I(Y_{i} \in (0, \Delta_{i}]) \right]^{\frac{G(\Delta_{i})}{n}}\times \\ && \exp\left(\frac{1}{n} \int_{\Delta_{i}}^{+\infty} g(s| \lambda(X_{i}), \eta)\log(g(s| \lambda(X_{i}), \eta))ds\right) \\ && \times \phi(X_{j}; \mu, \sigma^{2})^{\frac{1}{n}}\Bigg\}. \end{array} $$

Note that *E* (log(*L*_*T*_(***β***,*η*,*μ*,*σ*^2^))) = *E* (log(*ℓ*_*Y*_(***β***,*η*,*μ*,*σ*^2^))) when all samples involved are independent, which is mild because all observations in this paper are collected nationwide. It is easy to check that the maximum likelihood estimator based on (3) is asymptotically the same as that based on (1), which is consistent and satisfies the asymptotic normality under some general conditions.

However, *ℓ*_*Y*_(***β***,*η*,*μ*,*σ*^2^) contains some integral values

$$\begin{array}{@{}rcl@{}} \left\{\int_{\Delta_{i}}^{+\infty} g(s| \lambda(X_{i}), \eta)\log(g(s| \lambda(X_{i}), \eta))ds\right\}_{i=1}^{n}, \end{array} $$

which are computationally difficult. Fortunately, it holds that

$$\begin{array}{@{}rcl@{}} &&\frac{1}{n} \sum_{i=1}^{n} \int_{\Delta_{i}}^{+\infty} g(s| \lambda(X_{i}), \eta) \log(g(s|\lambda(X_{i}), \eta)) ds \\ &=& \frac{1}{n} \sum_{i=1}^{n} \left(\log(\eta)-\eta\log(\lambda(X_{i}))\right) \left(1-G(\Delta_{i}| \lambda(X_{i}), \eta)\right)\\ && - \frac{1}{n} \sum_{i=1}^{n}\left\{\left[\left(\frac{\Delta_{i}}{\lambda(X_{i})}\right)^{\eta} + 1\right] \exp\left(-\left(\frac{\Delta_{i}}{\lambda(X_{i})}\right)^{\eta}\right)\right\}\\ && + (\eta-1)\frac{1}{n} \sum_{i=1}^{n} \int_{\Delta_{i}}^{+\infty}\log(s)dG(s| \lambda(X_{i}), \eta). \end{array} $$

Noting that the estimation of *μ*,*σ*^2^ is trivial based on $\{X_{i}\}_{i=1}^{n}$, we focus on how to estimate ***β***, *η* in the sequel. To handle the integral values, we propose to use the EM algorithm as follows. Here suppose that we have obtained some initial estimators $\hat {\boldsymbol \beta }^{(0)}, \hat {\eta }^{(0)}$, which may be easily computed by pretending *Y*_*i*_’s having no bias.

- **E-step**. Set $\boldsymbol \beta ^{(k)} = \hat {\boldsymbol {\beta }}^{(0)}$, $\eta ^{(k)} = \hat {\eta }^{(0)}$. Generate *m*=1000 independent random numbers $\left \{u_{j}\right \}_{j=1}^{m}$, which is uniformly distributed over [*p*_min_,1), where *p*_min_= min{*p*_*i*_} with $p_{i} = G(\Delta _{i}| {\boldsymbol X}_{i}^{\top } \boldsymbol \beta ^{(k)}, \eta ^{(k)}) $. Put
  $$\begin{array}{@{}rcl@{}} \tilde{\text{\L}}_{Y}^{k}(\boldsymbol{\beta}, \eta) &:=& \frac{1}{n} \sum_{i=1}^{n} G(\Delta_{i}| {\boldsymbol X}_{i}^{\top} \boldsymbol{\beta}, \eta) \\ && \log\left\{g(Y_{i}| {\boldsymbol X}_{i}^{\top} \boldsymbol{\beta}, \eta)\right\} \\ & &I(Y_{i} \in (0, \Delta_{i}])\\ && + \frac{1}{n} \sum_{i=1}^{n} \left(\log(\eta)-\eta\log({\boldsymbol X}_{i}^{\top} \boldsymbol \beta)\right) \\ &&\left(1-G(\Delta_{i}| {\boldsymbol X}_{i}^{\top} \boldsymbol{\beta}, \eta)\right)\\ && - \frac{1}{n} \sum_{i=1}^{n}\left\{\left[\left(\frac{\Delta_{i}}{{\boldsymbol{X}}_{i}^{\top} \boldsymbol \beta}\right)^{\eta} + 1\right] \right.\\ && \left. \exp\left(-\left(\frac{\Delta_{i}}{{\boldsymbol{X}}_{i}^{\top} \boldsymbol \beta}\right)^{\eta}\right)\right\}\\ && + (\eta-1)\frac{1}{n} \sum_{i=1}^{n}\left\{(1-p_{i})\frac{1}{m_{i}}\sum_{u_{j}\geq p_{i}} \right. \\ && \left. \log \left(G^{-1}(u_{j}| {\boldsymbol X}_{i}^{\top} \boldsymbol \beta^{(k)}, \eta^{(k)}) \right) \right\}, \end{array} $$
  where ${\boldsymbol X}_{i}^{\top } = (1, X_{i}, X_{i}^{2}, X_{i}^{3})$, *G*^−1^(·) denotes the inverse function of *G*(·), and *m*_*i*_ is the cardinal number of the set {*j*:*u*_*j*_≥*p*_*i*_,*j*=1,2,···,*m*}.
- **M-step**. Maximize $\tilde {\text {\L }}_{Y}^{k}(\boldsymbol {\beta }, \eta)$ with respect to (***β***,*η*) and obtain the new estimators, namely, $\hat {\boldsymbol {\beta }}^{(k+1)}$, $\hat \eta ^{(k+1)}$. Check whether
  $$\begin{array}{@{}rcl@{}} \sum_{i=0}^{3} |\hat{\beta}_{i}^{(k+1)} - \hat{\beta}_{i}^{(k)}| + |\hat\eta^{(k+1)} - \hat\eta^{(k)}| < \frac{1}{n^{\delta}}, \\ \quad\text{for some } \delta>0.5. \end{array} $$
  If it is true, return $\hat {\boldsymbol {\beta }} = \left (\hat {\beta }_{0}^{(k+1)}, \hat {\beta }_{1}^{(k+1)}, \hat {\beta }_{2}^{(k+1)}, \hat {\beta }_{3}^{(k+1)}\right)^{\top }$ and $\hat \eta = \hat \eta ^{(k+1)}$; otherwise, repeat the **E-step** and **M-step** until convergence achieves.

We have coded this algorithm by **R** program relying on the optimization function *constrOptim()*. The implementation runs quite fast. An additional file shows the codes in detail (See Additional file 2). Usually, convergence can achieve by several iterations.

## Results

Based on the information of 2172 confirmed cases, we computed the estimated parameters by using the implementation of the EM algorithm mentioned above; see Table 1. It is worth mentioning that $\hat {\beta }_{3} = -1.1 \times 10^{-6}$ is very small, which implies that the cubic form *λ*_3_(*x*)=*β*_0_+*β*_1_*x*+*β*_2_*x*^2^+*β*_3_*x*^3^ is flexible enough to characterize the trend of the conditional mean *E*(*T*|*X*) on *X*. We do not need to assume a higher-order polynomial for *λ*(*x*).

**Table 1:** The results of parameter estimation

Density	Parameter	Estimate
*ϕ*(*x*;*μ*,*σ*^2^)	$\hat {\mu }$	43.15
	$\hat {\sigma }^{2}$	230.49
*g*(*y*|*λ*(*x*),*η*))	$\hat {\eta }$	1.81
	$\hat {\boldsymbol {\beta }}$	(10.24, −0.13, 2.0×10^−3^, −1.1×10^−6^)

**Fig. 4:**
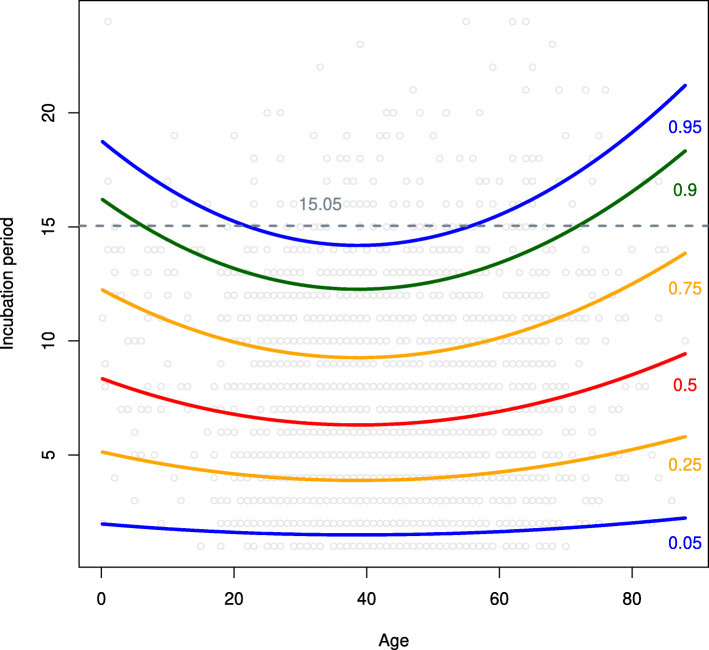
The estimated conditional quantiles of the incubation period distribution of COVID-19. 15.05 is the estimated 0.95-th quantile of the unconditional incubation periods

Using results in Table 1, we obtained the conditional 0.05, 0.25, 0.5, 0.75, 0.9, and 0.95-th quantiles of the incubation period distribution of COVID-19 on ages; see Fig. 4 for details.

Figure 4 indicates that quantiles corresponding to people of the middle ages seem to be less than those of the others. Especially, the estimated 0.95-th conditional quantile of the children and the elderly is greater obviously than that of the middle-aged. To be more detailed, we specify the 0.95-th conditional quantiles of the incubation period distribution of COVID-19 on different ages in Table 2. Table 2 indicates that the 0.95-th conditional quantiles of people in the age group 23 ∼55 lie between 14 and 15 days, shorter than those of the other groups. We also list the numbers of cases in each group and the corresponding proportions. It turns out the infected cases of age 23 ∼55 account for more than 70% of the total collected cases. Further, note that we collected 136 cases whose incubation periods are greater than 14 days. We provide the distribution of these cases over all age groups. It is shown that the age group 23 ∼55 accounts for the smallest proportion, only 5.11%, and the proportion of other age groups far exceeds 5%.

**Table 2:** Summary on the age groups divided according to the estimated 0.95-th quantile of the incubation period distribution of COVID-19, where $\tilde {n}_{j}$ denotes the number of infected cases in each age group, and $\tilde {m}_{j}$ the number of those cases with incubation period over 14 days in each age group

Item	Incubation period interval							
	14-15	15-16	16-17	17-18	18-19	19-20	20-21	21-22
Age group(s)	23 ∼55	15 ∼22	9 ∼14	4 ∼8	0∼3	80 ∼83	84 ∼86	≥88
		56 ∼63	64 ∼69	70 ∼74	75 ∼79			
Number $\tilde {n}_{j}$ in each group	1545	93	24	24	18	6	6	1
		232	156	45	22			
(total number *n*_*i*_)	(1545)	(325)	(180)	(69)	(40)	(6)	(6)	(1)
Proportion $\tilde {n}_{j} / n$	71.13%	4.28%	1.1%	1.1%	0.83%	0.28%	0.28%	0.05%
		10.68%	7.18%	2.07%	1.01%			
(total proportion *n*_*i*_/*n*)	(71.13%)	(14.96%)	(8.28%)	(3.17%)	(1.84%)	(0.28%)	(0.28%)	(0.05%)
Number $\tilde {m}_{j}$ in each group	79	3	3	1	3	1	1	0
		19	16	8	2			
(total number *m*_*i*_)	(79)	(22)	(19)	(9)	(5)	(1)	(1)	(0)
Proportion $\tilde {m}_{j} / n_{i}$	5.11%	3.23%	12.5%	4.17%	16.67%	16.67%	16.67%	0%
		8.19%	10.26%	17.78%	9.09%			
(total proportion *m*_*i*_/*n*_*i*_)	(5.11%)	(11.42%)	(22.76%)	(21.95%)	(25.76%)	(16.67%)	(16.67%)	(0%)

Furthermore, in order to further verify the results above, we divide the observed incubation periods into three groups by age: 0 to 25 years old, 26 to 60 years old, and over 60 years old, according to *World Population Prospects: the 2019 Revision*^1^. Then we fit the Weibull distribution in each group by setting *λ*(*x*)=*β*_0_.

**Fig. 5:**
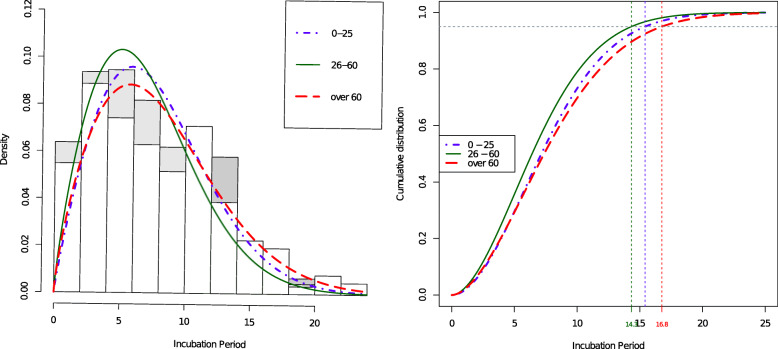
The fitted density and distribution functions of the incubation period for three age groups. The age groups are 0 ∼25, 26 ∼60, and over 60. The left indicates fitted density functions. The right indicates fitted distribution functions

Figure 5 shows that people under the age of 0 to 25 years old or over 60 years old have a higher probability that would emerge longer incubation period than people under the age of 26 to 60 years old. Moreover, the right figure is the fitted Weibull distribution function of the incubation period of COVID-19 in three age groups. It is obvious that the 0.95 quantile of people under 26 and over 60 is greater than that of people aged from 26 to 60. This roughly coincides with the results reported in Fig. 4, and hence indicates that the conditional distribution considered above can characterize the relationship of age and real incubation period of COVID-19.

## Discussion

Our estimation of the conditional quantiles indicates that the incubation period of COVID-19 varies depending on the age of the infected cases. Precisely, the incubation period of the young and the old tends to be longer than that of the middle-aged people.

It seems that we can find some supports from the immune theory in medical science. Note that human immunity refers to the sensitivity of the immune system in response to infection. During the incubation period, since the host’s immune system has not yet been activated, and the body has not begun to show symptoms, the virus can use this period to make a lot of replications. In many situations, to the infection, the more responsive the host’s immune system is, the shorter the incubation period tends to be. By further noting that the human immunity is weak at the beginning, improves with age, and will decline in the old period [23, 24], it hence maybe not surprised to see that the incubation period of the young and the old cases are longer than that of the middle-aged cases.

Currently, the quarantine duration is fixed to 14 days. It does not consider any other facts, e.g., age. Hence, our results may be helpful for disease control and prevention efforts, because it enables us to take some more precise measures. For example, personalized quarantine duration can be taken for individuals of different ages. Especially, people between the ages of 23 ∼55 play important roles in real life and are a significant part of the labor force. Besides, they account for the largest proportion of the population. A relatively short quarantine duration for them not only can reduce the burden of the medical staff but also is conducive to social-economic development. On the contrary, the conditional quantiles on ages 0 ∼22 and over 55 are much greater than 14 days. We may need to extend the quarantine duration for people of these ages. Such extension may help the prevention but have limit impacts on social-economic development.

It is worth mentioning there exist some other ways in statistics to characterize the conditional quantile of the incubation period over age. That is, first model the relationship between *T* and *X* by the following linear model:

$$\begin{array}{@{}rcl@{}} T = \beta_{0} + \beta_{1} X + \beta_{2} X^{2} + \beta_{3}X^{3} + \varepsilon, \end{array} $$

and then use the technique of quantile regression to estimate the unknown parameters *β*_*i*_,*i*=0,1,2,3. However, note that the true incubation period *T* cannot be fully observed, and the observed incubation period *Y* is randomly smaller than *T*. The estimated quantiles may suffer from some problem, e.g., underestimation.

**Fig. 6:**
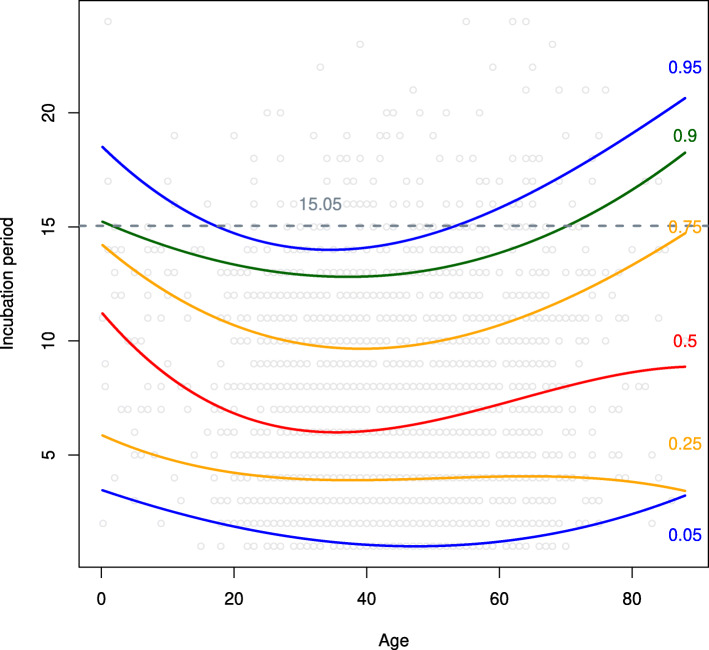
The estimated regression quantiles. The quantiles are 0.05, 0.25, 0.5, 0.75, 0.9, 0.95 from the bottom to top

In fact, we also report in Fig. 6 the result of the ordinary quantile regression mentioned above. Figure 6 shows that the regression quantiles follow a similar fashion to the conditional quantiles reported in Fig. 4. Nevertheless, we also note that the 0.25-th quantile and the 0.05-th quantile intersect with each other when the age is greater than 80. It seems difficult to have a reasonable explanation for this phenomenon. This strange result may be caused by the biased sampling issue. Hence, we did not take the regression quantiles to analyze the current data, although it provides some similar results as the conditional quantiles.

## Conclusion

In this paper, a model of the age effect on quantiles of the incubation period distribution of COVID-19 was proposed to explore the influence of age on the incubation period for COVID-19. Based on the collected data, our model showed that the incubation period of COVID-19 varies depending on the age of the infected cases. Specifically, the incubation period of the young and the old tends to be longer than that of middle-aged people. These findings enable us to take some more precise measures rather than fixed ones and thus may be helpful for disease control and prevention efforts. For example, personalized quarantine duration, namely shorter for middle-aged people and longer for the young and the old, can be taken for individuals of different ages, instead of fixed 14 days. People aged 23 ∼55 are a major part of the labor force and account for the largest proportion of the population, therefore, such methods may help the prevention but have limit impacts on social-economic development.

There are two major contributions in the papers. First, a relatively comprehensive description of the age effect on the incubation period of COVID-19 is provided. By modeling the conditional quantiles of the incubation period distribution given the age, we can learn about any quantiles of the incubation period of COVID-19 for people of certain ages. In contrast to methods that divide people into different age groups, it offers more information for selecting a more adequate quarantine period. Second, a reasonable likelihood function (3) was proposed to tackle the biased sampling problem when modeling the conditional distribution of the incubation period given age. The proposed likelihood function can almost eliminate the undesirable consequences and lead to a better estimation of the incubation period distribution of COVID-19.

It is worth mentioning that we are aware that some other researchers have also discussed the relationship between the age and the incubation period of COVID-19 during our submission. Some of them indicate that the age has no significant effect on the incubation period of COVID-19 [25, 26], or the length of the incubation period and age are positively correlated [27], while some others report that the age does have an effect [28–30]. However, most existing studies on the effect issue of age are summary, depending on a few discrete age subgroups. Besides, few of them touched on the biased sampling issue. Note that our current study takes age into account as a covariate. Hence, it can serve a purpose beyond the summary study.

Furthermore, although 2172 confirmed cases were included in the study, the data only covered confirmed cases through February 16th, 2020 in 29 provinces outside Hubei province in China. Since COVID-19 has spread worldwide, the effect pattern of age on the incubation period in different countries and regions needs further verification. How about the effect of some other factors, e.g., climatic conditions and air pollution status, on the incubation period of COVID-19 is also of great interest and worthy of further studies. However, it is beyond the scope of the current paper. We will pursue it in the future.

## Supplementary Information

**Additional file 1** 1. Variables description
Age: age of the confirmed case
InDate: infection date
OutDate: onset date
InPeriod: incubation Period
2. Values of variables
Age: the original age of the confirmed case
InDate and OutDate: for convenience, the values of InDate and OutDate were represented as the number of days departing from December 31, 2019, on which WHO announced the disease COVID-19 have become a public health incident. For example, the date January 1, 2020 takes the value 1.
Incubation Period: Onset date - Infection date + 1

**Additional file 2** 1. Data file path
To ensure that the program runs successfully, the data file “Additional file 1.xls” should be placed in the current working directory of R, which can be shown by running the code “getwd()” in **R**.
An alternative way is adding the file path in Line 18 of the code.
2. R packages needed
R packages including “readxl” “splines” “ggplot2” “quantreg” and “SparseM” should be successfully installed before running the program. All the packages can be installed by running “install.packages()”, such as “install.packages(“readxl”)” for package “readxl”.

## Data Availability

The data used and analysed during the current study and the source links are available from the additional files of this article (see Additional file 1).

## Abbreviations

WHO: World Health Organization
COVID-19: Coronavirus disease 2019
2019-nCoV: 2019-novel coronavirus
SARS-CoV: Severe acute respiratory syndrome coronavirus
SARS: Severe acute respiratory syndrome
MERS: Middle East respiratory syndrome

## Footnotes

1 https://population.un.org/wpp/
